# Synergistic bactericidal activity of a novel dual β-lactam combination against methicillin-resistant *Staphylococcus aureus*

**DOI:** 10.1093/jac/dkae165

**Published:** 2024-06-04

**Authors:** Hala Altarawneh, Turki Alhomra, Mohanned Alharbi, Yaxin Fan, Jeremy P Derrick, Guoqing Xia

**Affiliations:** Division of Evolution, Infection and Genomics, School of Biological Sciences, Faculty of Biology, Medicine and Health, University of Manchester, Manchester Academic Health Science Centre, Manchester M13 9PT, UK; Division of Evolution, Infection and Genomics, School of Biological Sciences, Faculty of Biology, Medicine and Health, University of Manchester, Manchester Academic Health Science Centre, Manchester M13 9PT, UK; Division of Evolution, Infection and Genomics, School of Biological Sciences, Faculty of Biology, Medicine and Health, University of Manchester, Manchester Academic Health Science Centre, Manchester M13 9PT, UK; Division of Evolution, Infection and Genomics, School of Biological Sciences, Faculty of Biology, Medicine and Health, University of Manchester, Manchester Academic Health Science Centre, Manchester M13 9PT, UK; Division of Evolution, Infection and Genomics, School of Biological Sciences, Faculty of Biology, Medicine and Health, University of Manchester, Manchester Academic Health Science Centre, Manchester M13 9PT, UK; Division of Evolution, Infection and Genomics, School of Biological Sciences, Faculty of Biology, Medicine and Health, University of Manchester, Manchester Academic Health Science Centre, Manchester M13 9PT, UK

## Abstract

**Objectives:**

MRSA is a major cause of hospital-acquired and community-acquired infections. Treatment options for MRSA are limited because of the rapid development of β-lactam resistance. Combining antibiotics offers an affordable, time-saving, viable and efficient approach for developing novel antimicrobial therapies. Both amoxicillin and cefdinir are oral β-lactams with indications for a wide range of bacterial infections and mild side effects. This study aimed to investigate the *in vitro* and *in vivo* efficacy of combining these two β-lactams against MRSA strains.

**Methods:**

Fourteen representative prevalent MRSA strains with diverse sequence types (STs) were tested with a combination of amoxicillin and cefdinir, using chequerboard and time–kill assays. The *Galleria mellonella* larvae infection model was used to evaluate the *in vivo* efficacy of this dual combination against the community-acquired MRSA (CA-MRSA) strain USA300 and the hospital-acquired MRSA (HA-MRSA) strain COL.

**Results:**

The chequerboard assay revealed a synergistic activity of the dual amoxicillin/cefdinir combination against all tested MRSA strains, with fractional inhibitory concentration index (FICI) values below 0.5 and at least a 4-fold reduction in the MICs of both antibiotics. Time–kill assays demonstrated synergistic bactericidal activity of this dual combination against the MRSA strain USA300 and strain COL. Moreover, *in vivo* studies showed that the administration of amoxicillin/cefdinir combination to *G. mellonella* larvae infected with MRSA strains significantly improved the survival rate up to 82%, which was comparable to the efficacy of vancomycin.

**Conclusions:**

*In vitro* and *in vivo* studies indicate that the dual combination of amoxicillin/cefdinir demonstrates a synergistic bactericidal efficacy against MRSA strains of various STs. Further research is needed to explore its potential as a treatment option for MRSA infections.

## Introduction

The emergence and spread of antimicrobial-resistant pathogens present a formidable challenge to human health, given that numerous infectious diseases caused by these pathogens have limited treatment options.^1^ Of principal concern, MRSA is one of the most common antimicrobial-resistant pathogens and is associated with a wide variety of severe human infections such as suppurative pneumonia, pyogenic endocarditis, sepsis and osteomyelitis.^2^ MRSA strains have become resistant to nearly all β-lactams. In addition, resistance to other antimicrobial classes, including macrolides, aminoglycosides, fluoroquinolones and tetracyclines, in some MRSA strains has further limited treatment options.^2^

Currently, invasive MRSA infections are primarily treated with vancomycin, daptomycin or linezolid.^3^ However, all these antibiotics have limitations, including toxicity and slower bacterial killing when compared with β-lactams.^4,5^ Additionally, the increased use of these antibiotics has led to the emergence of resistance.^6^ Therefore, there is an urgent need to develop novel anti-MRSA therapies.

In our recent efforts to identify new agents capable of reversing β-lactam resistance in MRSA, we conducted a screening of the FDA-approved drug library and discovered synergistic efficacy of a combination of amoxicillin and cefdinir active against the MRSA strain USA300. In this study, we report the *in vitro* and *in vivo* synergistic efficacy of this combination against diverse MRSA strains.

## Materials and methods

### Bacterial strains and culture conditions

Bacterial strains (Table S1, available as Supplementary data at *JAC* Online) were grown on tryptone soya agar (TSA) and TS broth (TSB) (Sigma–Aldrich, USA). Antibiotics (Merck KGaA, Germany) used in this study included amoxicillin, cefdinir and vancomycin.

### Antimicrobial susceptibility testing

The MICs of the antibiotics were evaluated using a broth microdilution method based on CLSI guidelines.^7^ Using cation-adjusted Mueller-Hinton broth (CAMHB), 2-fold serial dilutions of antibiotics were prepared and placed into 96-well microtitre plates (Corning, USA). Next, bacterial cultures were adjusted with saline to an OD_600_ value of 0.1, and then a volume of 5 µL of the prepared inoculum was added into each well of a 96-well microtitre plate. After incubating the plate at 37°C for 20 h, the MICs were determined using an Epoch2 microplate reader (BioTek^®^, UK).

### Chequerboard assay

Chequerboard assays were performed in triplicate in 96-well polystyrene microtitre plates (Corning Inc., Germany) to determine the combined effects of amoxicillin/cefdinir. The antibiotic stock solutions were subjected to serial 2-fold dilutions in CAMHB, starting with drug concentrations initially set at 2-fold higher than their respective MIC values. The serial dilution of the two antibiotics were then placed in a 96-well plate, followed by inoculation of each well with a volume of 5 μL of bacterial suspension at a final concentration of 1 × 10^6^ cfu/mL. After incubating the plates at 37°C for 20 h, the OD_600_ values of each well were measured using an Epoch2 microplate reader (BioTek^®^, UK).

The interaction of the two antibiotics was assessed by calculating the FIC index (FICI), using the following formula: FICI = (MIC of drug A in a combination/MIC of drug A alone) + (MIC of drug B in a combination/MIC of drug B alone). For MIC values that were not precisely determined, the next highest measurable concentration was recorded as the MIC value and used for calculating FIC and FICI. The MIC and FICI values are reported as median values. Synergy was described as an FICI of ≤0.5, an additive interaction with an FICI of 0.5–1, and indifference with an FICI of 1–4.^8^

### Time–kill assays

To evaluate the synergistic efficacy of amoxicillin and cefdinir against MRSA over time, time–kill assays were performed in triplicate for the community-acquired MRSA (CA-MRSA) strain USA300 and hospital-acquired MRSA (HA-MRSA) strain COL. Briefly, a volume of 10 mL of TSB was inoculated with an overnight bacterial culture to achieve a final concentration of 1 × 10^7^ cfu/mL. Subsequently, subcultures of the strain USA300 received a combination of 16 mg/L amoxicillin (^1^/_64_ MIC) and 1 mg/L cefdinir (^1^/_64_ MIC), as well as individual treatments with amoxicillin and cefdinir at their respective MICs. Similarly, the COL strain was exposed to a combination of 32 mg/L amoxicillin (^1^/_4_ MIC) with 1 mg/L cefdinir (^1^/_64_ MIC), along with individual amoxicillin and cefdinir treatments, each at the same concentration. Bacterial viability was assessed at 0, 2, 4, 6 and 24 h post-treatment by withdrawing aliquots from each tube, serially diluting them, and plating them onto TSA plates for colony counting. After incubation at 37°C for 24 h, colonies were enumerated, and the data were analysed using GraphPad Prism software (San Diego, CA, USA).

Bactericidal activity was defined as a reduction of ≥3 log_10_ cfu/mL over 24 h compared with the starting inoculum. Synergistic interaction of the dual antibiotic combination was defined as achieving a reduction of at least 2 log_10_ in cfu/mL compared with the most active single antibiotic after 24 h.^9^

### Resistance assay

To investigate the development of drug resistance, the MRSA strain USA300 was streaked onto TSA plates containing subinhibitory concentrations of amoxicillin (^1^/_2_ MIC) alone, cefdinir (^1^/_2_ MIC) alone, or a combination of amoxicillin (^1^/_128_ MIC) and cefdinir (^1^/_64_ MIC). Colonies were restreaked daily onto these three plates for 14 days. Subsequently, colonies were picked and subjected to MIC determination. These values were then compared with the MIC of the same MRSA strain prior to the antibiotic treatment, to assess any changes in antibiotic susceptibility.

### Galleria mellonella infection model

*G. mellonella* larvae (Livefoods Direct Limited, Sheffield) were employed as infection hosts to investigate the efficacy of the antibiotic combination against MRSA.^10^ Only larvae weighing 250 ± 50 mg and showing no signs of melanization or deformities were selected to ensure consistent dose administration and avoid sample biases. Larvae were stored in a cold, dark room and used within 1 week of receipt. The larvae were infected either with CA-MRSA strain USA300 or with HA-MRSA strain COL. Briefly, overnight bacterial cultures were diluted in sterile saline to an OD_600_ value of 0.3. Groups of 10 randomly selected larvae (*n* = 10/group) were infected with 5 μL of MRSA inoculums (5 × 10^6^ cfu/mL) using a 22-gauge Hamilton syringe (Fisher Scientific, UK) via the last right proleg of the larvae.

Two hours post-infection, each group of larvae received a single 2 μL injection into the last left proleg with one of the following treatments: amoxicillin at 30 mg/kg, cefdinir at 7 mg/kg, a combination of both amoxicillin (30 mg/kg) and cefdinir (7 mg/kg), or vancomycin at 5 mg/kg. These antibiotic dosages were chosen based on and comparable to the therapeutic dosages recommended for paediatric patients.^11,12^ A group of unmanipulated larvae and larvae treated with PBS were included as controls. Subsequently, the larvae were incubated at 37°C in sterile Petri and survival was monitored every 24 h for 7 days. All experiments were performed in triplicate. Mean survival rates were plotted using GraphPad Prism software. The final survival rates were compared using the log-rank (Mantel–Cox) test. Differences in survival rates between treatment groups were considered statistically significant if *P* ≤ 0.05.

## Results

### In vitro synergy of amoxicillin and cefdinir against MRSA

To study the interaction of amoxicillin and cefdinir in killing MRSA, MIC testing and chequerboard assays were performed against a panel of MRSA strains of various STs. As presented in Table 1, the MICs of amoxicillin ranged from 32 to >1024 mg/L, while those of cefdinir ranged from 1 to >256 mg/L.

**Table 1.** Median MIC (mg/L) and FICI values of amoxicillin (AMX) and cefdinir (CDR) against MRSA and MSSA strains

Strains	AMX MIC (mg/L)		CDR MIC (mg/L)		Median FICI (range)
	Alone	In combination^a^	Alone	In combination^a^	
MRSA					
BK1563	1024	32 (16–32)	4	0.5 (0.25–0.5)	0.156 (0.078–0.156)
BTN1823	>1024^c^	16	256	64	0.257
HT2002	256	16 (8–16)	1 (0.5–1)	0.031	0.093 (0.062–0.093)
MW2	1024	64	128	2	0.078
USA300	1024	16	64	2	0.047
USA700	256	1 (0.5–1)	8	1	0.128 (0.126–0.128)
57/92	>1024	128 (128–256)	>128	64	0.312 (0.312–0.375)
AO9973^b^	512	8	32	4	0.140
BTN2299	256	2	2 (1–2)	0.062	0.038 (0.038–0.070)
BTN766	512	64	256 (256–512)	8 (8–16)	0.156
COL	128	2	64	2	0.047
ON40899^b^	>1024	128 (64–128)	>256	4	0.070 (0.039–0.070)
ST398	256	16	16	2	0.188
W44646	32	8	32	1	0.281
MSSA					
BTN2289	128	32	128	8	0.313
C3	32	0.032	1 (0.5–1)	0.062	0.063 (0.063–0.125)
C427	32	1 (0.5–1)	0.125	0.016	0.159 (0.143–0.159)
D279	1024	2 (2–4)	0.125	0.016	0.129 (0.129–0.131)
D470	8 (4–8)	0.125 (0.063–0.125)	0.125	0.016	0.143
H399	1024	2	256	16 (8–16)	0.064 (0.033–0.064)

^a^The MICs of AMX and CDR in combination were determined in the presence of half the MIC of each other and reported as median values with their corresponding ranges.

^b^Sweden strains.

^c^For the MIC values that were not exactly determined, the next highest measurable concentration was recorded as MIC value and used for calculations of FIC and FICI.

The chequerboard assays demonstrated significant synergistic activity of amoxicillin and cefdinir against the MRSA strain USA300, as indicated by an FICI value of 0.047 (Table 1). Of note, strain USA300 exhibits high MICs for both amoxicillin and cefdinir. Remarkably, when combined at half the MIC of cefdinir, the amoxicillin MIC decreased from 1024 to 16 mg/L. Similarly, when combined at half the MIC of amoxicillin, the cefdinir MIC reduced from 64 to 2 mg/L. Similar synergistic activity between amoxicillin and cefdinir was observed in other MRSA strains, with an FICI value of less than 0.5 (Table 1). This suggests that the dual amoxicillin/cefdinir combination exhibits synergistic activity against MRSA strains of diverse backgrounds.

Additionally, synergistic activities of this dual combination were observed in six additional MSSA strains with FICI values less than 0.5 (Table 1), indicating that amoxicillin and cefdinir act synergistically against both MRSA and MSSA stains.

### Synergistic bactericidal activity of amoxicillin and cefdinir against MRSA

**Figure 1.**
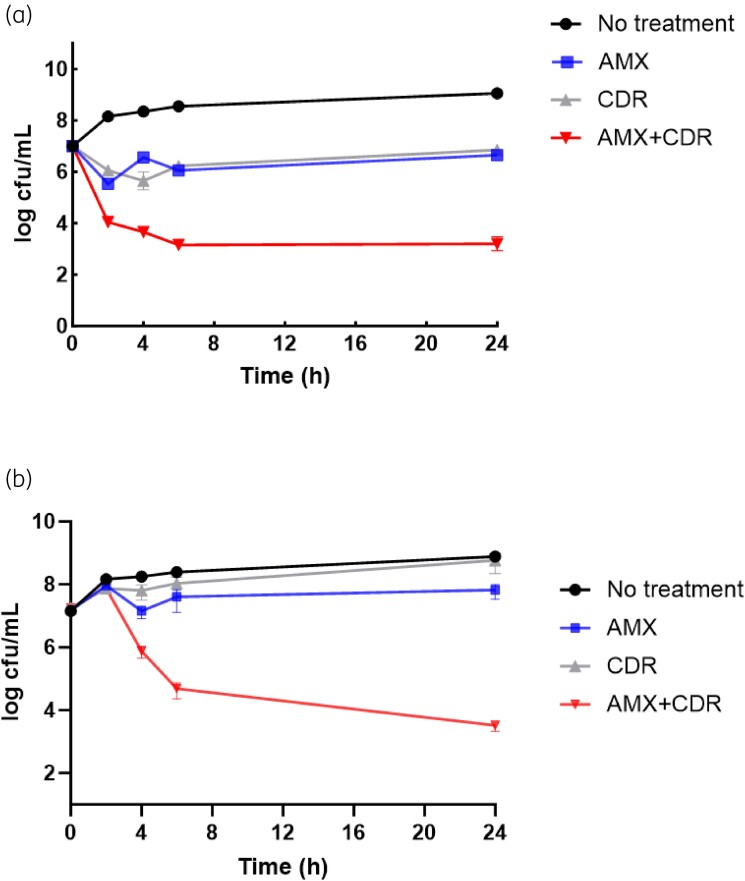
Time–kill curves. Time–kill analysis indicates synergistic bactericidal activities of the dual amoxicillin/cefdinir combination against the MRSA strain USA300 (a) and strain COL (b). Amoxicillin (AMX) and cefdinir (CDR), either alone or in combination, were added to log-phase cultures, and cfu counts were conducted at 0, 2, 4, 6 and 24 h. Strain USA300 received a combination of 16 mg/L AMX (^1^/_64_ MIC) and 1 mg/L CDR (^1^/_64_ MIC), as well as individual treatments with AMX and CDR at their respective MICs. Similarly, the COL strain was exposed to a combination of 32 mg/L AMX (^1^/_4_ MIC) with 1 mg/L CDR (^1^/_64_ MIC), along with individual AMX and CDR treatments, each at the same concentration. Data represent the means of three biological replicates.

Time–kill assays were conducted for the MRSA strains USA300 and COL. As shown in Figure 1(a), when amoxicillin or cefdinir was used alone, initial inhibition of USA300 growth was observed after 2 h, followed by bacterial regrowth, whereas the amoxicillin/cefdinir combination led to a reduction of >3 log_10_ after 24 h of incubation, indicating synergistic bactericidal activity. Similarly, the amoxicillin/cefdinir combination exhibited synergistic bactericidal activity against the MRSA strain COL (Figure 1b).

### Resistance development study

To investigate the potential development of antibiotic resistance, MRSA strain USA300 was serially passaged in a sublethal concentration of amoxicillin alone, cefdinir alone or in combination. After passaging for 14 days, we observed a 2-fold increase in the MICs of both antibiotics for MRSA isolates exposed to either cefdinir or amoxicillin alone. In contrast, the MICs of both antibiotics did not change for the MRSA isolates exposed to the dual amoxicillin/cefdinir combination.

### Efficacy of the dual amoxicillin/cefdinir combination in G. mellonella infection model

**Figure 2.**
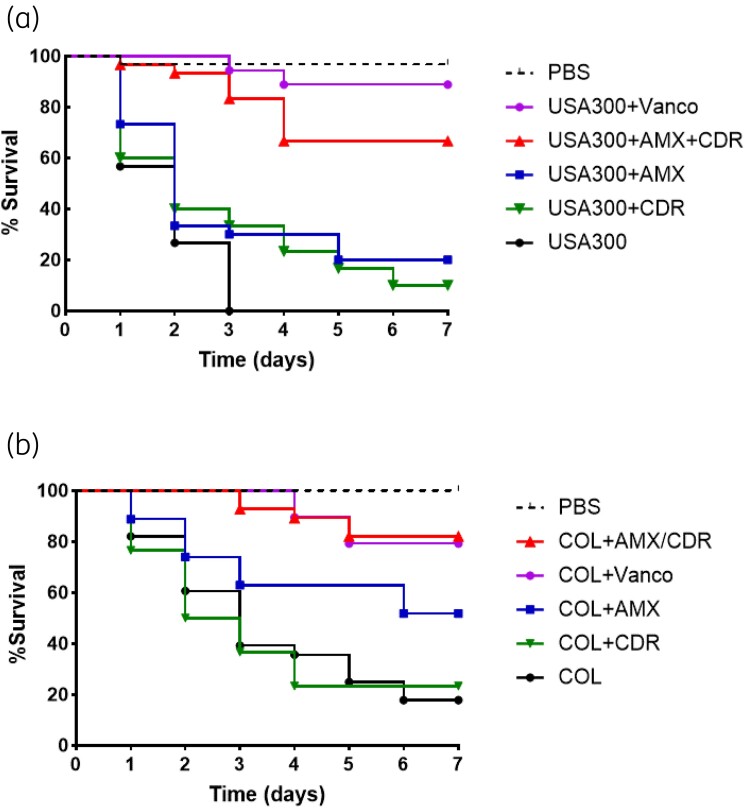
Survival curves of MRSA infected *G. mellonella* larvae. *G. mellonella* larvae were infected with the MRSA strain USA300 (a) or the strain COL (b) and treated with a single dose of amoxicillin (AMX), cefdinir (CDR) alone, or a combination of amoxicillin/cefdinir or vancomycin. The survival rates of the larvae in the different treatment groups were monitored for 7 days post-infection. Larvae in the uninfected group were treated with sterile PBS. Data represent the means of three independent experiments.

The *G. mellonella* model was used to assess the *in vivo* efficacy of the dual amoxicillin/cefdinir combination. The survival rate of the infected larvae after a single-dose antibiotic treatment was monitored for 7 days. As shown in Figure 2(a), strain USA300-infected larvae were killed within 3 days. When the larvae were treated with amoxicillin, only 20% of the larvae survived 7 days post-infection, and with cefdinir, the survival rate was 10%. In contrast, the survival rate of the larvae was improved to 67% at 7 days post-infection when treated with the dual amoxicillin/cefdinir combination, which is comparable to the efficacy of vancomycin (Figure 2a). Similarly, the survival rate of larvae infected with the MRSA strain COL was significantly improved to 82% when treated with the amoxicillin/cefdinir combination (Figure 2b). These findings suggest that this dual combination significantly increases MRSA killing in a *G. mellonella* infection model compared with the most active agent, amoxicillin.

## Discussion

In this study, we have characterized a novel dual β-lactam combination, amoxicillin/cefdinir, that exhibits synergistic bactericidal activity against MRSA strains of various STs. The chequerboard assay demonstrated a substantial reduction in the MICs for MRSA strains, achieving a minimum 4-fold decrease for both antibiotics when used in combination. In the time–kill assay, the combination resulted in a greater than 3 log_10_ reduction of the MRSA strain USA300 and the strain COL at 24 h post-treatment, clearly indicating synergistic bactericidal activity of the amoxicillin/cefdinir combination against MRSA strains. Importantly, the *G. mellonella* infection model demonstrates the superior *in vivo* efficacy of the dual amoxicillin/cefdinir combination compared with individual antibiotics.

Both amoxicillin and cefdinir have been explored in combination therapies for MRSA. Alou *et al.*^13^ demonstrated *in vitro* activity of amoxicillin/clavulanic acid with mupirocin against staphylococci, including MRSA. However, its *in vivo* efficacy remains unclear. Similarly, Ferrer-González *et al.*^14^ reported synergy between cefdinir and the FtsZ-targeting agent TXA707 against MRSA.

In a previous study, it was reported that a triple β-lactam combination meropenem/piperacillin/tazobactam acts synergistically and is bactericidal against MRSA strains.^15^ The underlying mechanisms involve the simultaneous disruption of multiple components of the MRSA cell-wall synthesis system, the inhibition of the essential transpeptidases PBP1 and PBP2 by meropenem and piperacillin, respectively, as well as the protection of piperacillin from β-lactamase degradation by tazobactam. Furthermore, meropenem induces allosteric changes in the active site of PBP2a, augmenting susceptibility to inhibition by another antibiotic molecule in the triple combination. In this study, we discovered the synergistic bactericidal activities of amoxicillin/cefdinir against MRSA strains. It was known that cefdinir specifically targets PBP2 and PBP3 while amoxicillin exhibits high affinity to PBP1, PBP2 and PBP3 in *S aureus*.^14,16^ The observed synergistic bactericidal activities are highly likely due to efficient inhibition of β-lactamase and PBPs by the amoxicillin/cefdinir combination in MRSA, resulting in enhanced bacterial killing.

Amoxicillin and cefdinir are two oral β-lactams with good pharmacokinetic properties that are widely used in clinical practice. Amoxicillin exhibits favourable pharmacokinetic properties, with a high bioavailability ranging from 77% to 93%, and effectively penetrates various tissues.^17^ In paediatric patients, it can reach up to 73.6 mg/L in plasma at a dose of 25 mg/kg twice daily.^18^ Pharmacokinetic studies show cefdinir concentrations in blister fluid remain equal to or exceed plasma levels for 6–12 h after administration, with cefdinir exposure in blister fluid/plasma ranging from 92.4% to 108.4%.^19^ Peak free plasma concentrations of amoxicillin and cefdinir reached up to 60 and 1 mg/L, respectively, based on their protein binding rates of 17% and 60%.^20,21^ Additionally, amoxicillin and cefdinir are broad-spectrum antibiotics used to treat infections caused by Gram-positive and Gram-negative bacteria, such as MSSA, streptococci and *Haemophilus influenzae*.^20,22^ They are prescribed for respiratory tract infections, skin and soft tissue infections, and urinary tract infections.^20,22^ In this study, the dual combination demonstrated synergistic activity against MRSA strains in time–kill assays and in the *G. mellonella* larvae infection model at clinically achievable concentrations, suggesting its potential for treating MRSA infections.

In conclusion, our data suggest that the dual amoxicillin/cefdinir combination represents a promising treatment option for MRSA infections, such as skin and soft tissue infections, with potential for broader clinical use. Further validation in a skin and soft tissue infection model is needed to validate the efficacy of this dual combination. Moreover, although we observed a 2-fold increase in the MIC of amoxicillin or cefdinir in a 14-day resistance assay, longer-term studies involving more clinical strains are required to fully elucidate the potential resistance development.

## Supplementary Material

dkae165_Supplementary_Data

## Supplementary data

Table S1 is available as Supplementary data at *JAC* Online.
