# Supplementary material for: Synergistic bactericidal activity of a novel dual β-lactam combination against methicillin-resistant Staphylococcus aureus
Source: J Antimicrob Chemother. 2024 Jun 4;79(7):1677–82. doi: 10.1093/jac/dkae165 (PMC11215534; doi:10.1093/jac/dkae165)
Supplement: dkae165_Supplementary_Data [file dkae165_supplementary_data.docx]

**Supplementary Table S1:** A list of bacterial strains used in this study.

| **Strains** | **Description** | **References** |
| --- | --- | --- |
| 57/92 | HA-MRSA, ST239 | (1) |
| AO9973* | HA-MRSA, ST22-A2 | (2) |
| BK1563 | CA-MRSA, ST88 | (1) |
| BTN1823 | CA-MRSA, ST8 | (3) |
| BTN2289 | MSSA, ST5 | (4) |
| BTN2299 | HA-MRSA, ST45 | (4) |
| BTN766 | HA-MRSA, ST36 | (4) |
| C101 | MSSA, ST22 | (5) |
| C3 | MSSA, ST51 | (5) |
| C427 | MSSA, ST42 | (5) |
| COL | HA-MRSA | (6) |
| D279 | MSSA, ST25 | (7) |
| D470 | MSSA, ST207 | (7) |
| H399 | MSSA, ST33 | (5) |
| HT2002-0664 | CA-MRSA, ST80 | (8) |
| MW2 | CA-MRSA, ST1 | (9) |
| ON408/99* | HA-MRSA, ST246 | (2) |
| ST398 | LA-MRSA | (10) |
| USA300 | CA-MRSA, ST8 | (11) |
| USA700 | CA-MRSA, ST72 | (12) |
| W44646 | *mecC* MRSA | (13) |

* Sweden strains.

**References:**

1. Enright MC, Robinson DA, Randle G, Feil EJ, Grundmann H, Spratt BG. The evolutionary history of methicillin-resistant Staphylococcus aureus (MRSA). Proc Natl Acad Sci U S A. 2002;99(11):7687-92.

2. Cookson BD, Robinson DA, Monk AB, Murchan S, Deplano A, de Ryck R, et al. Evaluation of molecular typing methods in characterizing a European collection of epidemic methicillin-resistant Staphylococcus aureus strains: the HARMONY collection. J Clin Microbiol. 2007;45(6):1830-7.

3. Lina G, Durand G, Berchich C, Short B, Meugnier H, Vandenesch F, et al. Staphylococcal chromosome cassette evolution in Staphylococcus aureus inferred from ccr gene complex sequence typing analysis. Clin Microbiol Infect. 2006;12(12):1175-84.

4. Price J, Baker G, Heath I, Walker-Bone K, Cubbon M, Curtis S, et al. Clinical and Microbiological Determinants of Outcome in Staphylococcus aureus Bacteraemia. Int J Microbiol. 2010;2010:654858.

5. Enright MC, Day NP, Davies CE, Peacock SJ, Spratt BG. Multilocus sequence typing for characterization of methicillin-resistant and methicillin-susceptible clones of Staphylococcus aureus. J Clin Microbiol. 2000;38(3):1008-15.

6. Shafer WM, Iandolo JJ. Genetics of staphylococcal enterotoxin B in methicillin-resistant isolates of Staphylococcus aureus. Infect Immun. 1979;25(3):902-11.

7. Feil EJ, Cooper JE, Grundmann H, Robinson DA, Enright MC, Berendt T, et al. How clonal is Staphylococcus aureus? J Bacteriol. 2003;185(11):3307-16.

8. Valle J, Toledo-Arana A, Berasain C, Ghigo J-M, Amorena B, Penadés JR, et al. SarA and not σB is essential for biofilm development by Staphylococcus aureus. Molecular Microbiology. 2003;48(4):1075-87.

9. Baba T, Takeuchi F, Kuroda M, Yuzawa H, Aoki K, Oguchi A, et al. Genome and virulence determinants of high virulence community-acquired MRSA. Lancet. 2002;359(9320):1819-27.

10. Larsen J, Petersen A, Larsen AR, Sieber RN, Stegger M, Koch A, et al. Emergence of Livestock-Associated Methicillin-Resistant Staphylococcus aureus Bloodstream Infections in Denmark. Clinical Infectious Diseases. 2017;65(7):1072-6.

11. Moran GJ, Krishnadasan A, Gorwitz RJ, Fosheim GE, McDougal LK, Carey RB, et al. Methicillin-resistant S. aureus infections among patients in the emergency department. N Engl J Med. 2006;355(7):666-74.

12. McDougal LK, Steward CD, Killgore GE, Chaitram JM, McAllister SK, Tenover FC. Pulsed-field gel electrophoresis typing of oxacillin-resistant Staphylococcus aureus isolates from the United States: establishing a national database. J Clin Microbiol. 2003;41(11):5113-20.

13. Kriegeskorte A, Ballhausen B, Idelevich EA, Köck R, Friedrich AW, Karch H, et al. Human MRSA isolates with novel genetic homolog, Germany. Emerg Infect Dis. 2012;18(6):1016-8.
